# Prognostic Value of the Systemic Immune-Inflammation Index (SII) for short-term clinical outcomes in the emergency department: a retrospective cohort study

**DOI:** 10.1186/s12873-026-01613-9

**Published:** 2026-05-12

**Authors:** Mehmet Şirin Büyükkaya, Osman Taş, Mahmut Şahin, Mehmet Yorgun, Ramazan Sami Aktaş

**Affiliations:** Department of Emergency Medicine, Van Training and Research Hospital, University of Health Sciences, Van, Turkey

**Keywords:** Systemic immune-inflammation index, Emergency department, Triage, Hospitalisation, Clinical deterioration, NEWS2, Early warning score, Neutrophil-to-lymphocyte ratio, Inflammatory biomarkers

## Abstract

**Background:**

The Systemic Immune-Inflammation Index (SII = PLT × NE# / LY#) is a composite haematological biomarker derivable from a routine complete blood count (CBC) that integrates neutrophil, platelet, and lymphocyte counts into a single value reflecting systemic inflammatory status.

**Objective:**

Studies simultaneously evaluating SII across multiple concurrent outcomes in an unselected ED population remain limited. We aimed to evaluate SII’s independent prognostic value for hospitalisation, ICU admission, clinical deterioration, in-hospital mortality, and 72-hour ED return, and its incremental value over NEWS2, NLR, and PLR.

**Methods:**

Retrospective cohort study of 6,739 adults presenting to a tertiary ED (January 2022–December 2023) with a CBC at presentation. SII, NLR, PLR, and NEWS2 were calculated from admission data. Primary outcome was hospitalisation; secondary outcomes were ICU admission, clinical deterioration, in-hospital mortality, and 72-hour ED return. Multivariable logistic regression and ROC analysis were performed.

**Results:**

Median SII was 768.1 (IQR: 442–1,532); 22.6% were hospitalised. SII independently predicted hospitalisation (aOR = 1.672, 95% CI: 1.551–1.803, *p* < 0.001; per one ln-unit increase in SII, equivalent to approximately a 2.7-fold increase in raw SII value); however, standalone discriminative performance was modest (AUC = 0.640) and should not be interpreted as clinically actionable in isolation. The primary clinical contribution was incremental: adding SII to NEWS2 increased the AUC from 0.717 to 0.758 (DeLong *p* < 0.001), with both markers retaining independent significance after mutual adjustment. Discriminative performance for secondary outcomes was limited (ICU admission AUC 0.430; clinical deterioration AUC 0.561; 72-hour return AUC 0.581), and SII did not independently predict in-hospital mortality after adjustment (AUC 0.571; *p* = 0.222).

**Conclusion:**

The principal contribution of SII in this study is its incremental prognostic value when combined with NEWS2, rather than standalone discrimination. SII may serve as a practical adjunct to established early warning scores during ED triage. Prospective multicentre validation is required before clinical implementation.

## Introduction

Triage and early clinical decision-making in the emergency department are pivotal determinants of patient safety and efficient resource allocation [1, 2]. Risk stratification conducted within the first hour of presentation — encompassing the likelihood of hospital admission, intensive care, and clinical deterioration — is particularly consequential [3, 4]. Accordingly, the identification of biomarkers that are immediately available, cost-neutral, and interpretable at the bedside has become a leading research priority in emergency medicine [5, 6].

In acute illness, the interplay between systemic inflammation and host immune defence represents a fundamental mechanism that determines clinical outcomes.4 Neutrophil activation, platelet-mediated coagulo-inflammatory cascades, and lymphocyte-driven adaptive immunity are the three principal components of this axis; disruption of any one is associated with organ dysfunction and adverse outcomes [5, 6]. Conventional biomarkers such as C-reactive protein (CRP) and procalcitonin capture this interplay in a unidimensional fashion and do not reflect critical processes such as platelet activation.6 By contrast, haematological indices derived from routine CBC offer the potential to encode all three components in a single value at no additional cost.

The neutrophil-to-lymphocyte ratio (NLR) and platelet-to-lymphocyte ratio (PLR) are first-generation haematological inflammation indices with established prognostic value, yet NLR omits platelet activation and PLR incompletely captures neutrophil-driven inflammation [7, 8]. To address these limitations, Hu et al. introduced the Systemic Immune-Inflammation Index (SII = PLT × NE# / LY#) in 2014 [9], simultaneously encoding all three components. Originally validated in hepatocellular carcinoma, SII has since shown independent associations with mortality in sepsis (J-shaped dose-response relationship, MIMIC-IV cohort [10]), ICU patients [11], and general populations [12, 13]. In emergency medicine, Islam et al. noted SII’s accessibility while cautioning against standalone use [8].

Despite this growing body of evidence, existing SII studies have focused predominantly on disease-specific populations (sepsis, myocardial infarction, stroke, oncology). Relatively few studies have evaluated SII across multiple concurrent outcomes in a general ED population [7, 8]. Moreover, most published studies employ a single endpoint, omit comparative ROC analysis against NLR and PLR, and do not report on the association between paradoxically low SII values and adverse outcomes in the most critically ill.

We evaluated the independent prognostic value of SII across four concurrent clinical outcomes in 6,739 unselected ED adults. To our knowledge, few studies have simultaneously assessed SII against multiple outcomes in a general ED population with direct comparison against NEWS2. Our primary hypothesis was that admission SII independently predicts short-term ED outcomes and may serve as a complementary triage adjunct.

## Methods

### Study design and ethical approval

This was a retrospective, observational, single-centre cohort study of adult patients presenting to the ED of a Van Training and Research Hospital, University of Health Sciences, Van, Turkey between January 2022 and December 2023. Data were retrieved from the hospital information management system (HIMS). The study was conducted in accordance with the Declaration of Helsinki and ICH-GCP guidelines. Ethics committee approval was granted by the Van Training and Research Hospital Ethics Committee (Protocol No: GOKAEK/2026-1-20, 2026). Given the retrospective design and use of anonymised data, the requirement for individual informed consent was waived by the committee.

### Study population

Eligible patients were adults (≥ 18 years) presenting between January 2022 and December 2023 with a CBC obtained within 60 min of registration (T0-CBC ≤ 60 min). Patients were excluded if any of the following criteria were met: age < 18 years; trauma-related presentation (blunt, penetrating, or polytrauma); haematological malignancy (leukaemia, lymphoma, or myeloproliferative disease); active chemotherapy or radiotherapy; immunosuppressive or corticosteroid therapy; erythrocyte or platelet transfusion within 24 h prior to presentation; confirmed pregnancy (where documented in the hospital information system); known haematological disorder associated with severe thrombocytopenia or thrombocytosis; missing or erroneous CBC records; or duplicate registration (index visit retained). Trauma patients were specifically excluded because acute haemorrhage and haemodilution markedly alter neutrophil, platelet, and lymphocyte counts independently of the immune-inflammatory response, rendering SII unreliable in this context. Together, these exclusions were designed to preserve the biological validity of SII as an immune-inflammatory biomarker and are consistent with the pre-registered study protocol (Ethics Committee Protocol No: GOKAEK/2026-1-20). Data extraction was performed by two independent investigators (M.Ş.B. and O.T.); discrepancies were resolved by consensus with a third investigator (M.Ş.) and verified against source records in a 10% random sample.

### Data collection

The following variables were extracted from the HIMS: demographics (age, sex); vital signs at presentation (systolic and diastolic blood pressure [mmHg], heart rate [bpm], respiratory rate [/min], peripheral oxygen saturation [SpO₂, %], temperature [°C], Glasgow Coma Scale [GCS]); laboratory values (CBC: PLT [×10³/µL], NE# [×10³/µL], LY# [×10³/µL]; SII [PLT × NE#/LY#], NLR [NE#/LY#], and PLR [PLT/LY#] were calculated from these); comorbidities (diabetes mellitus [DM], hypertension [HT], coronary artery disease [CAD], chronic obstructive pulmonary disease [COPD]); triage category (colour code); time variables (ED length of stay [ED-LOS, minutes] and T0-CBC [minutes]); and presenting diagnosis (ICD-10 code). The National Early Warning Score 2 (NEWS2) was calculated from the admission vital signs using the Royal College of Physicians scoring system, incorporating respiratory rate, oxygen saturation, systolic blood pressure, heart rate, level of consciousness (GCS), and temperature.

### SII calculation

SII was calculated using the formula originally described by Hu et al. [9]:

$$\eqalign{ {\rm{SII = }} & {\rm{PLT}}\left( {{\rm{ \times 10/\mu L}}} \right){\rm{ \times }} \cr& {\rm{NE\# }}\left( {{\rm{ \times 10/\mu L}}} \right) \cr & {\rm{/LY\# }}\left( {{\rm{ \times 10/\mu L}}} \right) \cr} $$

The three components reflect distinct pathophysiological processes: NE# encodes the innate immune response to acute inflammation and tissue injury; PLT reflects coagulo-inflammatory activation and endothelial dysfunction; and LY# represents adaptive immune capacity and the degree of immune suppression [10]. Cases with LY# = 0 (*n* = 0) were excluded to prevent mathematical singularity. Given the pronounced right skew of the SII distribution, natural log-transformed SII [ln(SII)] was used in all regression analyses.

### Outcomes

#### Primary outcome

Hospitalisation following the ED visit (ward or ICU admission; coded 0 = discharged, 1 = admitted).

**Secondary outcomes**

i ICU admission: Direct ICU admission or transfer from the ward to the ICU (disposition code 2).
ii Clinical deterioration: Among hospitalised patients, the composite occurrence of non-invasive mechanical ventilation (NIMV), vasopressor or inotropic support, endotracheal intubation, or in-hospital death (clinical outcome codes 1–4). Discharge with recovery served as the comparator (code 0).
iii 72-hour ED return visit: Any unplanned return to the same ED within 72 h of the index visit (coded 0 = no, 1 = yes; cases coded 9 [unassessable] were excluded from this analysis).
iv In-hospital mortality: Death occurring during the index hospitalisation, among patients admitted to a ward or ICU (coded as clinical outcome 4). This outcome was assessed in the hospitalised subset only.

### Statistical analysis

Data were analysed using Python 3.11 (SciPy 1.17, scikit-learn 1.8). Normality was assessed with the Shapiro–Wilk test. As SII and the other haematological indices deviated substantially from normality, non-parametric tests were applied throughout. Two-group comparisons were performed using the Mann–Whitney U test; multi-group comparisons used the Kruskal–Wallis test with post-hoc pairwise Mann–Whitney U corrections. Categorical variables were compared using the chi-squared test. Correlations between SII and continuous clinical variables were quantified with Spearman’s rank correlation coefficient (rs).

Independent predictors were identified by univariable and multivariable logistic regression (covariates: age, sex, SBP, SpO₂, DM, HT); results are reported as adjusted odds ratios (aOR) with 95% confidence intervals (CI). SII was natural log-transformed [ln(SII)] prior to entry into all logistic regression models to account for its pronounced right skew. The resulting aORs represent the change in odds per one-unit increase in ln(SII), which corresponds approximately to a 2.72-fold (e-fold) increase in raw SII (e.g., from the cohort median of 768 to ~ 2,088). This scale should be considered when interpreting the reported ORs; a one ln-unit increase represents a clinically substantial rise in SII rather than a single-unit increment. Biomarker performance was compared by ROC analysis with Youden-optimal thresholds. The AUC difference between NEWS2-alone and NEWS2 + SII models was formally tested using the DeLong method. Internal validation of the combined model was assessed using bootstrap resampling (1,000 iterations). Formal calibration plots were not generated and represent a limitation. All subgroup and threshold analyses are exploratory. *p* < 0.05 (two-sided) was significant. Post-hoc power analysis confirmed adequate powering: the observed effect required a minimum of 601 patients for 80% power (α = 0.05); our *n* = 6,739 achieved power > 99.9%, with an events-per-variable ratio of 250.

## Results

### Demographic and clinical characteristics

A total of 6,739 patients were included. The median age was 52 years (IQR: 34–70) and 55.8% were female. Baseline demographic and clinical characteristics are presented in Table 1.

**Table 1:** Baseline demographic and clinical characteristics (*n* = 6,739)

Variable	Value	*n* / %
**Total patients**	6,739	100%
Age, median (IQR), years	52 (34–70)	—
Age, mean ± SD, years	52.2 ± 20.7	—
Sex – Female	3,763	55.8%
Sex – Male	2,976	44.2%
SII, median (IQR)	768.1 (442–1,532)	—
SII, mean ± SD	1,363.9 ± 1,917.7	—
NLR, median (IQR)	3.27 (1.97–6.64)	—
PLR, median (IQR)	139.7 (96.9–212.0)	—
PLT, mean ± SD, ×10³/µL	239.3 ± 81.0	—
NE#, mean ± SD, ×10³/µL	6.56 ± 3.72	—
LY#, mean ± SD, ×10³/µL	1.85 ± 2.39	—
ED-LOS, median (IQR), min	236 (175–299)	—
T0-CBC, median (IQR), min	15 (11–19)	—
Hypertension	1,294	19.2%
Coronary artery disease	701	10.4%
Diabetes mellitus	366	5.5%
COPD	52	0.8%

Hypertension was the most prevalent comorbidity (19.2%), followed by CAD (10.4%) and DM (5.5%). The median T0-CBC was 15 min (IQR: 11–19), confirming contemporaneous SII measurement [14].

### SII distribution and comorbidity analysis

Median SII was 768.1 (IQR: 442.4–1,532.2; mean ± SD: 1,363.9 ± 1,917.7), reflecting pronounced right skew. SII differed significantly across comorbidity groups (CAD, COPD, and HT all *p* ≤ 0.003; DM *p* = 0.525). Positive correlations were found with age (rs = 0.127) and heart rate (rs = 0.028), and a negative correlation with SpO₂ (rs = − 0.068; all *p* < 0.05).

### SII by disposition group

**Fig. 1:**
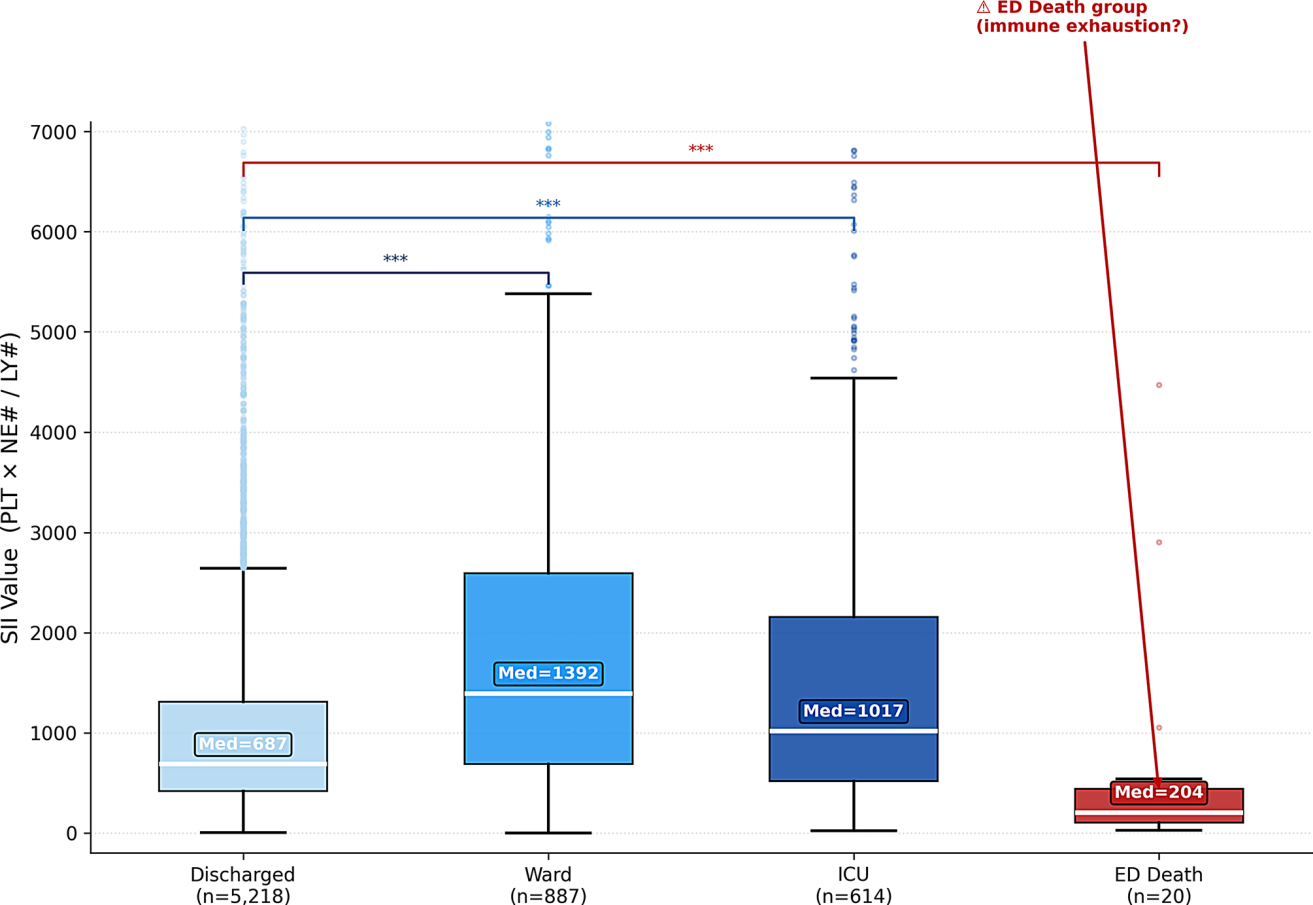
SII distribution by disposition group. Kruskal–Wallis H = 342.25, *p* < 0.001; all pairwise comparisons *p* < 0.001. The ED Death subgroup (*n* = 20) is a small-sample observation and should be interpreted with caution. ICU, intensive care unit; SII, systemic immune-inflammation index

Of 6,739 patients, 5,218 (77.4%) were discharged, 887 (13.2%) admitted to a ward, 614 (9.1%) to the ICU, and 20 (0.3%) died in the ED. SII differed significantly across all disposition groups (Kruskal–Wallis H = 342.25, *p* < 0.001): median SII was 687.3 in discharged, 1,391.7 in ward admissions, 1,016.9 in ICU admissions, and 203.7 in ED deaths. Notably, patients who died in the ED had the lowest median SII of all groups (203.7); given the small group size (*n* = 20), this observation should be interpreted cautiously. (Fig. 1)

### Clinical deterioration and 72-hour return visits

Among hospitalised patients (*n* = 1,501), clinical deterioration occurred in 274 (18.3%), with a significantly higher median SII than those with recovery (1,459.0 vs. 1,145.0; *p* = 0.002); however, discriminative performance was limited (AUC = 0.561). For 72-hour ED return visits, 113 patients who returned had significantly higher median SII than the 5,105 who did not (897.1 vs. 682.6; *p* = 0.003; AUC = 0.581). For both endpoints, the statistically significant group differences should be interpreted alongside the modest AUC values, which indicate limited standalone discriminative capacity.

### In-hospital mortality

Among the 1,501 hospitalised patients (ward or ICU admissions), 156 (10.4%) died during the index admission. Patients who died had a significantly higher median SII than survivors (1,536.4 [IQR: 729.4–3,390.7] vs. 1,166.7 [IQR: 594.8–2,348.5]; *p* = 0.004). However, SII achieved an AUC of only 0.571 for in-hospital mortality — lower than NLR (AUC = 0.620) and not meaningfully different from chance in this subgroup. In multivariable logistic regression adjusted for age, systolic blood pressure, and SpO₂, SII did not reach independent significance as a predictor of in-hospital mortality (aOR = 1.135, 95% CI: 0.917–1.392, *p* = 0.222; model AUC = 0.725). In-hospital mortality therefore represents a negative finding for SII as an independent predictor.

### ROC analysis

**Fig. 2:**
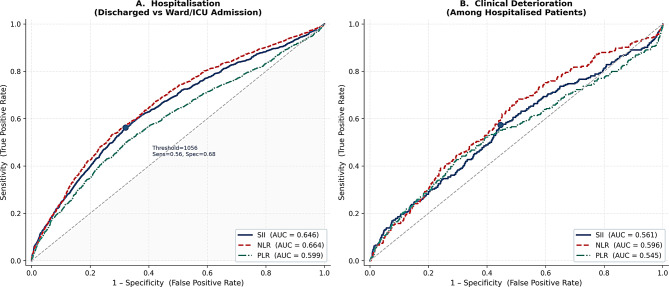
ROC curves for SII, NLR, and PLR. Panel **A**: Prediction of hospitalisation (SII AUC = 0.646, NLR AUC = 0.664, PLR AUC = 0.599). Panel **B**: Prediction of clinical deterioration among hospitalised patients (SII AUC = 0.561, NLR AUC = 0.616). AUC, area under the curve; NLR, neutrophil-to-lymphocyte ratio; PLR, platelet-to-lymphocyte ratio; SII, systemic immune-inflammation index

For hospitalisation, SII achieved an AUC of 0.646 (threshold: 1,055.7; sensitivity 56.2%, specificity 67.9%), comparable to NLR (AUC = 0.664) and superior to PLR (AUC = 0.599). SII AUC for 72-hour return was 0.581 and for clinical deterioration 0.561. The AUC for ICU admission was not statistically significant (0.430). (Fig. 2) Results are presented in Table 2.

**Table 2:** ROC analysis results for SII, NLR, and PLR across all outcomes

Outcome / Marker	AUC	95% CI	Threshold	Sensitivity	Specificity
Hospitalisation – SII	0.646	0.632–0.660	1,055.7	56.2%	67.9%
Hospitalisation – NLR	0.664	0.650–0.678	—	—	—
Hospitalisation – PLR	0.599	0.584–0.614	—	—	—
Hospitalisation – NE#	0.647	—	—	—	—
ICU admission – SII	0.430	—	NS	—	—
72-h ED return – SII	0.581	—	631.9	67.3%	46.3%
Clinical deterioration – SII	0.561	—	1,325.6	—	—

### Comparison with NEWS2

The National Early Warning Score 2 (NEWS2) was calculated for all 6,739 patients (median 2, IQR: 1–3). NEWS2 was significantly higher in hospitalised patients than in those discharged (median 3 [IQR: 1–4] vs. 1 [IQR: 0–2]; *p* < 0.001). For prediction of hospitalisation, NEWS2 alone achieved an AUC of 0.717 (95% CI: 0.700–0.733; optimal threshold: 3; sensitivity 57.4%, specificity 81.9%), outperforming SII alone (AUC = 0.640). When SII was added to NEWS2 in a combined logistic regression model, the AUC increased to 0.758 (95% CI: 0.744–0.774; Δ = +0.042 vs. NEWS2 alone); this improvement was statistically significant by DeLong test (Z = 9.87, *p* < 0.001) and confirmed by bootstrap internal validation (1,000 iterations). A model additionally incorporating age and sex achieved an AUC of 0.782 (Δ = +0.065 vs. NEWS2 alone). Hosmer–Lemeshow calibration of the combined model yielded χ²(8) = 51.35 (*p* < 0.001); this result should be interpreted cautiously in large samples (*n* = 6,739), where the test has high power to detect minor deviations from perfect calibration that may not be clinically meaningful. SII remained an independent predictor of hospitalisation after adjustment for NEWS2 (aOR = 1.683, 95% CI: 1.574–1.801, *p* < 0.001), and NEWS2 remained independently significant after adjustment for SII (aOR = 2.889, 95% CI: 2.630–3.228, *p* < 0.001), confirming complementary prognostic information.

### Multivariable logistic regression: hospitalisation

After adjustment for age, sex, systolic blood pressure, SpO₂, DM, and HT, SII remained an independent predictor of hospitalisation (aOR = 1.672, 95% CI: 1.551–1.803, *p* < 0.001; Nagelkerke R² = 0.382; model AUC = 0.800).

### Multivariable logistic regression: clinical deterioration

In the hospitalised subset, SII independently predicted clinical deterioration (aOR = 1.163, 95% CI: 1.020–1.325, *p* = 0.024; model AUC = 0.702).

### Quartile analysis

**Fig. 3:**
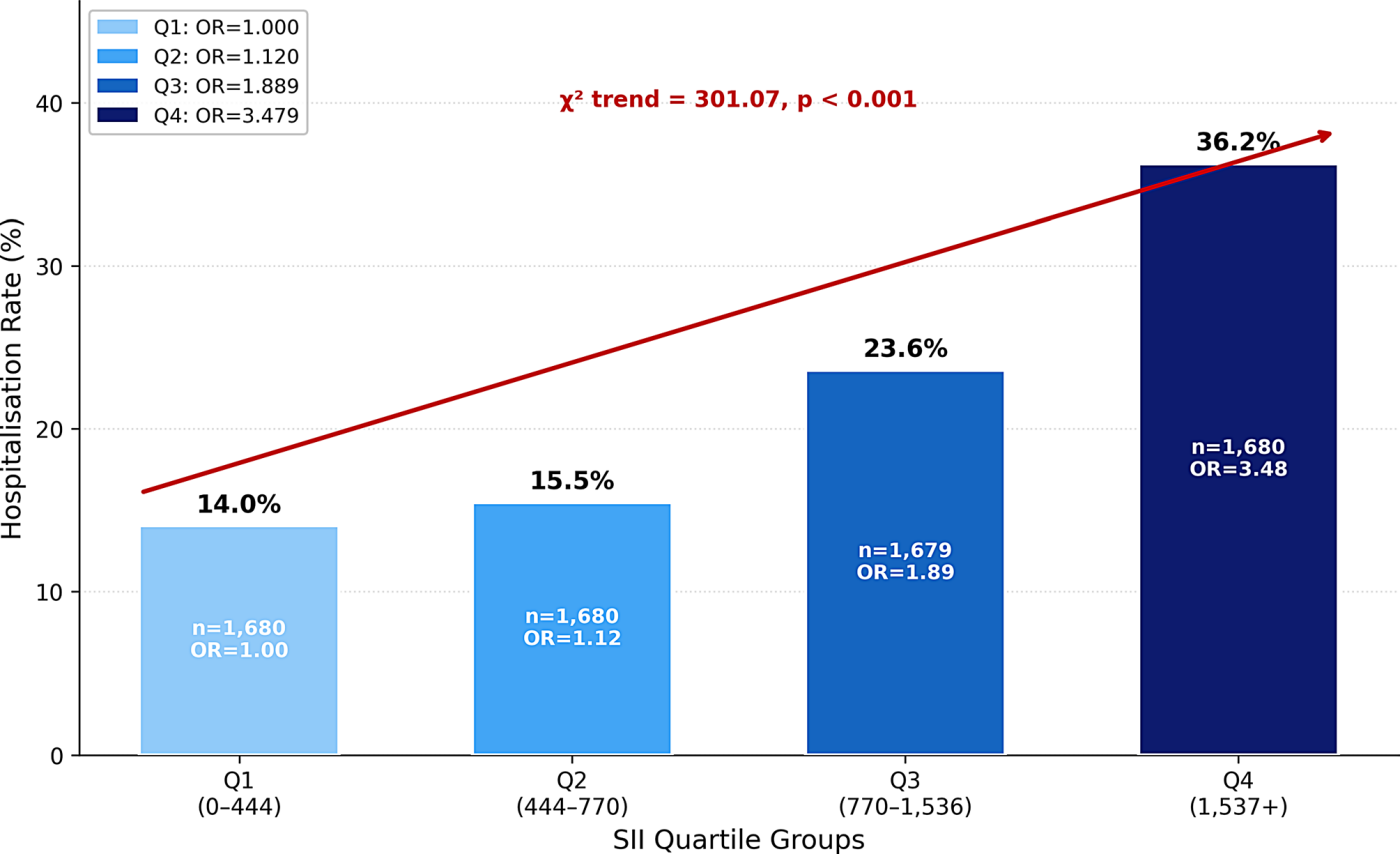
Hospitalisation rates and odds ratios by SII quartile (Q1–Q4). χ² trend *p* < 0.001. Error bars represent 95% CIs. OR, odds ratio; SII, systemic immune-inflammation index

Quartile analysis showed a significant dose-response relationship (χ² trend = 301.07, *p* < 0.001; Table 3). Q4 (SII ≥ 1,537) carried an approximately 3.5-fold higher hospitalisation risk vs. Q1 (OR = 3.479, 95% CI: 2.936–4.123). No significant difference was observed between Q1 and Q2, suggesting a floor effect below SII ~ 770. (Fig. 3)

**Table 3:** Hospitalisation rates and odds ratios by SII quartile

Quartile	SII Range	*n*	Hosp. Rate	OR (95% CI)
Q1 (ref)	0–444	1,680	14.0%	1.000 (reference)
Q2	444–770	1,680	15.5%	1.120 (0.926–1.356)
Q3	770–1,536	1,679	23.6%	1.889 (1.581–2.256)
Q4	≥ 1,537	1,680	36.2%	3.479 (2.936–4.123)

χ² trend = 301.07, *p* < 0.001

### Subgroup analysis

**Fig. 4:**
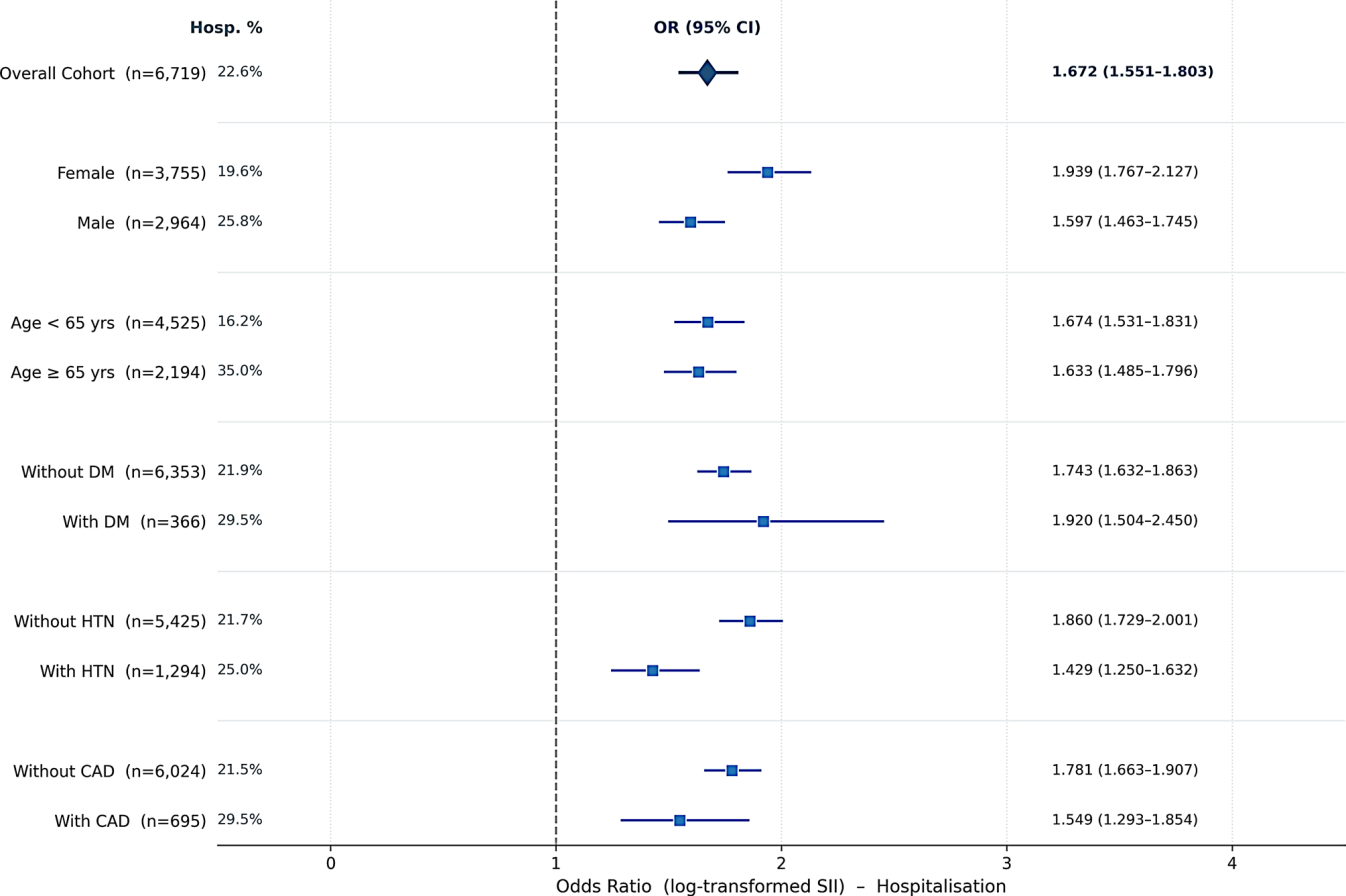
Forest plot of SII odds ratios for hospitalisation by subgroup. All subgroups: *p* < 0.001. CAD, coronary artery disease; CI, confidence interval; DM, diabetes mellitus; HT, hypertension; OR, odds ratio; SII, systemic immune-inflammation index

The following subgroup analyses are exploratory; they were not prespecified, carry risk of type I error due to multiple comparisons, and should not be used to guide clinical decisions without external validation. SII independently predicted hospitalisation across all prespecified subgroups (all *p* < 0.001), with the strongest association in patients with DM (OR = 1.920, AUC = 0.680) and modest attenuation in those with HT (OR = 1.429, AUC = 0.596); these differences are hypothesis-generating. (Fig. 4)

### SII distribution by triage category

**Fig. 5:**
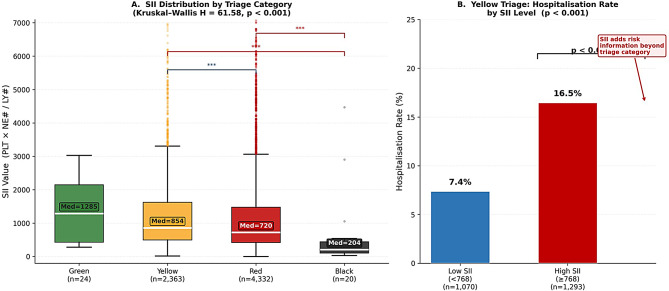
SII distribution by triage category and added prognostic value within Yellow triage. Panel A: Box plots of SII by triage category (Green *n* = 24, Yellow *n* = 2,363, Red *n* = 4,332, Black *n* = 20). Kruskal–Wallis H = 61.58, *p* < 0.001. Significant pairwise comparisons are indicated (*** *p* < 0.001). Panel B: Hospitalisation rates among Yellow-coded patients stratified by SII level (SII ≥ 768 vs. < 768, using the overall cohort median as threshold). Patients with elevated SII had more than twice the hospitalisation rate (16.5% vs. 7.4%, *p* < 0.001). SII AUC for hospitalisation: within Yellow triage = 0.665, within Red triage = 0.657. AUC, area under the curve; SII, systemic immune-inflammation index

SII differed significantly across triage categories (Kruskal–Wallis H = 61.58, *p* < 0.001). Median SII was lowest in the Black group (203.7) and highest in the Yellow group (853.7). Significant pairwise differences were observed between Yellow–Red, Yellow–Black, and Red–Black (all *p* < 0.001). (Fig. 5)

Within the Yellow triage subgroup, patients with SII ≥ 768 had more than twice the hospitalisation rate of those with SII < 768 (16.5% vs. 7.4%, *p* < 0.001; AUC = 0.665 within Yellow, 0.657 within Red), demonstrating that SII provides prognostic information complementary to assigned triage category.

### Diagnostic performance: PPV, NPV, and likelihood ratios

At the optimal threshold of 1,055.7, SII yielded a PPV of 33.5% and NPV of 84.4% for hospitalisation (LR + 1.75, LR − 0.64). These values should be interpreted cautiously: NPV is prevalence-dependent, and an LR − of 0.64 represents only a modest shift in post-test probability that is unlikely to be clinically actionable in isolation. The low PPV of 33.5% confirms SII cannot rule in hospitalisation. These diagnostic indices support the characterisation of SII as a risk-contextualisation tool rather than a standalone decision instrument.

### Age-stratified SII analysis

The following age-stratified analyses are exploratory and hypothesis-generating; age-specific thresholds have not been externally validated. Median SII was higher in patients aged ≥ 65 years (953.8; IQR: 516–2,042) than in younger groups (695.2 and 684.6, respectively; all *p* < 0.001), paralleling higher hospitalisation rates (35.0% vs. 12.2% and 21.8%). Optimal SII thresholds varied by age group (956 for < 45; 918 for 45–64; 1,120 for ≥ 65 years), suggesting a single universal threshold may be suboptimal — though this observation requires prospective age-stratified validation before any clinical application. (Table 4).

**Table 4:** Age-stratified SII distribution, hospitalisation rates, and optimal diagnostic thresholds

Age Group	*n*	SII Median (IQR)	Hosp. %	AUC	Opt. Threshold	NPV
< 45 years	2,643	695.2 (414–1,296)	12.2%	0.653	956	92.3%
45–64 years	1,882	684.6 (421–1,384)	21.8%	0.614	918	83.7%
≥ 65 years	2,194	953.8 (516–2,042)	35.0%	0.633	1,120	74.0%

Optimal thresholds determined by Youden Index. NPV calculated at group-specific prevalence. Kruskal–Wallis across age groups: H = 98.4, *p* < 0.001

### SII by presenting diagnosis

The following diagnosis-specific analyses are exploratory and hypothesis-generating; disease-specific thresholds have not been validated in independent cohorts. SII differed significantly across the eight most common presenting diagnoses (Kruskal–Wallis H = 393.26, *p* < 0.001). Highest median SII values were seen in pneumonia (1,264.7), dyspnoea (1,163.3), and abdominal pain (997.7); lowest in chest pain (534.1) and dizziness (578.7). Discriminative performance varied by diagnosis (dizziness AUC = 0.706; abdominal pain AUC = 0.692). Optimal thresholds ranged from 488 (dizziness) to 1,233 (dyspnoea); this marked variation suggests that a universal threshold may not be appropriate across diagnoses, though disease-specific thresholds require prospective validation before clinical use [15, 16].

## Discussion

### Principal findings and clinical implications

The principal finding of this study is that admission SII independently predicts hospitalisation across a large, diagnostically heterogeneous ED cohort (aOR = 1.672, 95% CI: 1.551–1.803, *p* < 0.001). A significant dose-response relationship was confirmed by quartile analysis (χ² trend *p* < 0.001), with patients in Q4 (≥ 1,537) facing approximately 3.5-fold greater odds of hospitalisation than those in Q1. Within triage categories, SII further refined risk: among Yellow-coded patients, those with SII ≥ 768 had more than twice the hospitalisation rate of those with SII < 768 (16.5% vs. 7.4%, *p* < 0.001). Evidence for secondary outcomes was mixed: SII was associated with clinical deterioration (*p* = 0.002) and 72-hour return (*p* = 0.003), though standalone discriminative performance was limited (AUC 0.561 and 0.581). For ICU admission and in-hospital mortality, SII should be considered a negative finding (AUC 0.430 and 0.571 respectively; mortality not independently significant, *p* = 0.222). Diagnostic index analysis yielded PPV 33.5% and NPV 84.4% (LR + 1.75; LR − 0.64); the modest LR− does not support standalone rule-out use. SII is derivable from routine CBC at no additional cost with median availability of 15 min; it should be considered a complementary risk-contextualisation tool rather than a standalone triage decision instrument.

### Incremental value of SII over NEWS2

The combination of NEWS2 and SII increased the AUC from 0.717 to 0.758 (95% CI: 0.744–0.774), confirmed by DeLong test (Z = 9.87, *p* < 0.001) and bootstrap resampling (1,000 iterations). Both markers retained independent significance after mutual adjustment, confirming that SII encodes inflammatory information not captured by vital signs alone. Hosmer–Lemeshow calibration testing indicated suboptimal fit (χ²(8) = 51.35, *p* < 0.001); in large samples such as ours (*n* = 6,739), this test has very high power to detect clinically trivial deviations, and the result should not be interpreted as clinically important miscalibration. Visual calibration plots and external validation remain necessary.

### Comparison with existing literature

Our findings are broadly concordant with the existing SII literature. The cohort median SII (768.1) closely matches the threshold identified by Jiang et al. in MIMIC-IV [10], and the Turkish ICU study corroborates SII’s independent prognostic signal in critically ill patients [11, 17–21]. Notably, Tekeli et al. reported an SII AUC of 0.646 for complicated appendicitis [22] — numerically identical to ours for hospitalisation — suggesting SII’s prognostic capacity is broadly consistent across diverse populations. NLR marginally outperformed SII for hospitalisation prediction (AUC 0.664 vs. 0.646), consistent with prior reports [23]; however, this difference is unlikely to be clinically meaningful, and SII retained independent predictive value in multivariable modeling [23, 24]. The platelet component gives SII an advantage over NLR in capturing platelet-driven inflammatory activation, particularly relevant in thrombocytopenia or thrombocytosis. PLR showed the lowest discriminative performance, suggesting limited standalone utility in a heterogeneous ED population. SII occupies a complementary niche to dynamic perfusion markers in ED risk stratification. Recent studies have demonstrated that perfusion-based markers such as early lactate dynamics and lactate clearance independently predict short-term mortality in ED sepsis cohorts [25, 26]. Unlike lactate, SII encodes the immune-inflammatory axis and is derivable from routine CBC; these marker classes are complementary rather than competing, and may serve different roles within early triage frameworks.

### SII in the ED death subgroup: a small-sample observation

Among disposition groups, patients who died in the ED had the lowest median SII (203.7; *n* = 20). This small-sample observation (0.3% of the cohort) should be interpreted with considerable caution. It may reflect haematological derangement in end-stage critical illness, but causality cannot be inferred from this retrospective dataset. This finding is strictly hypothesis-generating.

Among hospitalised patients, 156 (10.4%) died during the index admission. For in-hospital mortality, SII should be considered a negative finding in this study. Although median SII was significantly higher in non-survivors than survivors (1,536 vs. 1,167; *p* = 0.004), the AUC of 0.571 was not meaningfully different from chance, and SII did not independently predict mortality after multivariable adjustment (aOR = 1.135, *p* = 0.222). NLR outperformed SII for this endpoint (AUC 0.620 vs. 0.571). This pattern is consistent with the known limitations of single inflammatory indices for mortality prediction in heterogeneous hospitalised populations, where clinical severity markers dominate the prognostic signal.

SII independently predicted hospitalisation across all prespecified subgroups (all *p* < 0.001), with the strongest association observed in patients with diabetes mellitus (OR = 1.920, AUC = 0.680) and modest attenuation in those with hypertension (OR = 1.429, AUC = 0.596).

### Strengths

The principal strengths of this study are: (i) a large sample size (*n* = 6,739) providing adequate statistical power; (ii) simultaneous evaluation of four clinical outcomes, a breadth unusual in the SII literature; (iii) a documented T0-CBC interval (median 15 min) confirming contemporaneous SII measurement; (iv) comparative ROC analysis against NLR and PLR; and (v) formal DeLong testing and bootstrap internal validation (1,000 iterations) of the incremental AUC gain from combining SII with NEWS2.

### Limitations

This study has several important limitations. First, the retrospective, single-centre design precludes causal inference and limits generalisability to other healthcare settings, patient populations, and acuity profiles; multicentre prospective validation is required [27]. The requirement for a CBC at presentation introduced selection bias: patients too critically ill to have blood drawn were not captured, and this may have underestimated SII’s association with the most severe outcomes. Second, only a single SII time-point was available; serial measurements would better characterise the dynamic inflammatory trajectory. Third, although bootstrap internal validation (1,000 iterations) confirmed AUC stability and DeLong testing confirmed the significance of the incremental AUC gain (Z = 9.87, *p* < 0.001), the Hosmer–Lemeshow calibration test indicated suboptimal fit (χ²(8) = 51.35, *p* < 0.001). As noted above, this result may reflect the high statistical power of the HL test in large samples rather than clinically meaningful miscalibration; nevertheless, visual calibration plots and external prospective validation are required to confirm clinical reliability across risk strata. Fourth, formal documentation of exclusion criteria was not retrievable from the registry, and the 72-hour return endpoint was unassessable in 22.6% of cases. Fifth, the multivariable models were restricted to prespecified covariates (age, sex, SBP, SpO₂, DM, HT) to limit overfitting risk; important variables such as presenting diagnosis severity and full comorbidity burden (e.g., Charlson Comorbidity Index) were not included. Sixth, the deliberate inclusion of a diagnostically heterogeneous cohort limits the interpretability of findings for any specific disease group; the reported associations may differ substantially across diagnostic subgroups, and disease-specific validation in high-prevalence ED presentations (e.g., sepsis, pneumonia, acute coronary syndrome) is a priority for future work. Future research priorities include: prospective multicentre trials evaluating SII-informed triage; integrated early warning models combining SII with NEWS2 and perfusion markers; serial SII measurements in critically ill patients; and disease-specific SII validation in high-prevalence ED presentations.

## Conclusion

The principal contribution of this study is the demonstration that SII provides statistically significant incremental prognostic value over NEWS2 (DeLong *p* < 0.001) in an unselected adult ED population — a finding that may have potential clinical relevance given that both markers are available within minutes of presentation at no additional cost. SII also independently predicted hospitalisation across all prespecified subgroups, with patients in the highest quartile (Q4, ≥ 1,537) facing an approximately 3.5-fold greater risk. Standalone discriminative performance was modest (AUC 0.640–0.646 for hospitalisation; lower for secondary outcomes), and these findings should be interpreted cautiously given the retrospective single-centre design and absence of formal model calibration.

SII should not be used as a standalone triage decision tool. Its value lies in complementing — not replacing — established early warning scores such as NEWS2. Prospective multicentre validation of the combined NEWS2 + SII model, with formal calibration and assessment of clinical decision impact, represents the priority research agenda.

## Data Availability

Data are available upon reasonable request to the corresponding author.

## References

[1] Richardson DB. Increase in patient mortality at 10 days associated with emergency department overcrowding. Med J Aust. 2006;184(5):213–6. 16515430 10.5694/j.1326-5377.2006.tb00204.x

[2] Bernstein SL, Aronsky D, Duseja R, et al. The effect of emergency department crowding on clinically oriented outcomes. Acad Emerg Med. 2009;16(1):1–10. 19007346 10.1111/j.1553-2712.2008.00295.x

[3] Seymour CW, Gesten F, Prescott HC, et al. Time to treatment and mortality during mandated emergency care for sepsis. N Engl J Med. 2017;376(23):2235–44. 28528569 10.1056/NEJMoa1703058 PMC5538258

[4] Vincent JL, Marshall JC, Namendys-Silva SA, et al. Assessment of the worldwide burden of critical illness: the ICON audit. Lancet Respir Med. 2014;2(5):380–6. 24740011 10.1016/S2213-2600(14)70061-X

[5] Singer M, Deutschman CS, Seymour CW, et al. The Third International Consensus Definitions for Sepsis and Septic Shock (Sepsis-3). JAMA. 2016;315(8):801–10. 26903338 10.1001/jama.2016.0287 PMC4968574

[6] Seymour CW, Liu VX, Iwashyna TJ, et al. Assessment of clinical criteria for sepsis (Sepsis-3). JAMA. 2016;315(8):762–74. 26903335 10.1001/jama.2016.0288 PMC5433435

[7] Hwang SY, Shin TG, Jo IJ, et al. Neutrophil-to-lymphocyte ratio as a prognostic marker in critically ill septic patients. Am J Emerg Med. 2017;35(2):234–9. 27806894 10.1016/j.ajem.2016.10.055

[8] Islam MM, Satici MO, Eroglu SE. Unraveling the clinical significance and prognostic value of the NLR, PLR, SII, SIRI, and delta neutrophil index. Turk J Emerg Med. 2024;24(1):8–19. 38343523 10.4103/tjem.tjem_198_23 PMC10852137

[9] Hu B, Yang XR, Xu Y, et al. Systemic immune-inflammation index predicts prognosis of patients after curative resection for hepatocellular carcinoma. Clin Cancer Res. 2014;20(23):6212–22. 25271081 10.1158/1078-0432.CCR-14-0442

[10] Jiang D, Bian T, Shen Y, Huang Z. Association between admission systemic immune-inflammation index and mortality in critically ill patients with sepsis: a retrospective cohort study based on MIMIC-IV. Clin Exp Med. 2023;23(7):3641–50. 36930382 10.1007/s10238-023-01029-w PMC10022570

[11] Emgin O, Kilic ER, Taskiran I, Haftaci E, Ata A, Yilmaz M. Systemic Inflammation Index (SII) as a Predictor of Mortality in Intensive Care Units. Biomedicines. 2025;13(7):1669. 40722740 10.3390/biomedicines13071669 PMC12292411

[12] Yang YL, Wu CH, Hsu PF, et al. Systemic immune-inflammation index predicts clinical outcome in patients with coronary artery disease. Eur J Clin Invest. 2020;50(9):e13230. 32291748 10.1111/eci.13230

[13] Xia Y, Xia C, Wu L, et al. Systemic immune inflammation index (SII), system inflammation response index (SIRI) and risk of all-cause mortality and cardiovascular mortality: a 20-year follow-up cohort study of 42,875 US adults. J Clin Med. 2023;12(3):1128. 36769776 10.3390/jcm12031128 PMC9918056

[14] Pines JM, Hilton JA, Weber EJ, et al. International perspectives on emergency department crowding. Acad Emerg Med. 2011;18(12):1358–70. 22168200 10.1111/j.1553-2712.2011.01235.x

[15] Fois AG, Paliogiannis P, Scano V, et al. The systemic inflammation index on admission predicts in-hospital mortality in COVID-19 patients. Molecules. 2020;25(23):5725. 33291581 10.3390/molecules25235725 PMC7731255

[16] Zhao N, Di B, Xu LL. The NLR, PLR, LMR and SII in the prognosis of intracranial hemorrhage: a systematic review and meta-analysis. Clin Neurol Neurosurg. 2021;205:106622. 10.1016/j.clineuro.2021.106622. PMID: 33957447.

[17] Liu C, Wu X, Deng R, Xu X, Chen C, Wu L et al. Systemic immune-inflammation index combined with quick sequential organ failure assessment score for predicting mortality in sepsis patients. Heliyon. 2023;9(9):e19526. 10.1016/j.heliyon.2023.e19526. PMID: 37809490. 10.1016/j.heliyon.2023.e19526 PMC10558729 37809490

[18] Xu F, Zhang S, Zhang Y. High level of systemic immune inflammation index elevates delirium risk among patients in intensive care unit. Sci Rep. 2024;14(1):30265. 10.1038/s41598-024-81559-9. PMID: 39632969. 10.1038/s41598-024-81559-9 PMC11618353 39632969

[19] Tang Z, Liao C, Zhuang Z, et al. Trajectories of SII and mortality risk in moderate-to-severe traumatic brain injury. Front Neurol. 2025;15:1439318. 40012842 10.3389/fneur.2024.1439318 PMC11860105

[20] Efgan MG, Acar H, Kanter E, Kirik S, Duman Sahan T. Role of systemic immune inflammation index, systemic immune response index, neutrophil lymphocyte ratio and platelet lymphocyte ratio in predicting peritoneal culture positivity and prognosis in cases of spontaneous bacterial peritonitis admitted to the emergency department. Medicina. 2024;60(8):1335. 10.3390/medicina60081335. PMID: 39202616. 10.3390/medicina60081335 PMC11356178 39202616

[21] Gao F, Wang L, Liu J et al. Systemic immune-inflammation index and the short-term mortality of patients with sepsis: a meta-analysis. Biomol Biomed. 2025;25(4):798–809. 10.17305/bb.2024.11494. PMID: 39959392. 10.17305/bb.2024.11494 PMC11959392 39739368

[22] Tekeli A, Caliskan MB, Bahadir GB. Evaluation of SII efficacy in predicting complicated appendicitis in the paediatric ED. Ulus Travma Acil Cerrahi Derg. 2023;29(5):566–573. 10.14744/tjtes.2022.42472. PMID: 37145053. 10.14744/tjtes.2022.42472 PMC10277329 37145053

[23] Wang RH, Wen WX, Jiang ZP et al. The clinical value of neutrophil-to-lymphocyte ratio (NLR), systemic immune-inflammation index (SII), platelet-to-lymphocyte ratio (PLR) and systemic inflammation response index (SIRI) for predicting the occurrence and severity of pneumonia in patients with intracerebral haemorrhage. Front Immunol. 2023;14:1115031. 10.3389/fimmu.2023.1115031. PMID: 36860868. 10.3389/fimmu.2023.1115031 PMC9969881 36860868

[24] Demirpolat MT, Islam MM. The role of neutrophil-to-lymphocyte ratio, platelet-to-lymphocyte ratio, and systemic immune inflammation index in predicting the necessity for surgery and therapeutic surgery in patients with anterior abdominal stab wounds. World J Surg. 2024;48(6):1315–1322. 10.1002/wjs.12177. PMID: 38570898. 10.1002/wjs.12177 38570898

[25] Şengüldür E, Demir MC. Early lactate dynamics for short-term mortality in emergency department sepsis. BMC Emerg Med. 2025;25(1):213. 41131467 10.1186/s12873-025-01391-w PMC12548108

[26] Senguldur E, Demir MC, Selki K. Is Lactate Clearance Useful in Predicting Cardiopulmonary Resuscitation Outcome and 48-Hour Mortality? J Coll Physicians Surg Pak. 2025;35(3):267–73. 40055156 10.29271/jcpsp.2025.03.267

[27] Bernstein SL, Asplin BR. Emergency department crowding: old problem, new solutions. Emerg Med Clin North Am. 2006;24(4):821–37. 16982341 10.1016/j.emc.2006.06.013

